# Echolocation in Oilbirds and swiftlets

**DOI:** 10.3389/fphys.2013.00123

**Published:** 2013-05-28

**Authors:** Signe Brinkløv, M. Brock Fenton, John M. Ratcliffe

**Affiliations:** ^1^Department of Biology, Western UniversityLondon, ON, Canada; ^2^Institute of Biology, University of Southern DenmarkOdense, Denmark

**Keywords:** Oilbird, *Steatornis caripensis*, swiftlets, *Aerodramus*, *Collocalia*, echolocation, biosonar, click

## Abstract

The discovery of ultrasonic bat echolocation prompted a wide search for other animal biosonar systems, which yielded, among few others, two avian groups. One, the South American Oilbird (*Steatornis caripensis*: Caprimulgiformes), is nocturnal and eats fruit. The other is a selection of diurnal, insect-eating swiftlets (species in the genera *Aerodramus* and *Collocalia*: Apodidae) from across the Indo-Pacific. Bird echolocation is restricted to lower frequencies audible to humans, implying a system of poorer resolution than the ultrasonic (>20 kHz) biosonar of most bats and toothed whales. As such, bird echolocation has been labeled crude or rudimentary. Yet, echolocation is found in at least 16 extant bird species and has evolved several times in avian lineages. Birds use their syringes to produce broadband click-type biosonar signals that allow them to nest in dark caves and tunnels, probably with less predation pressure. There are ongoing discrepancies about several details of bird echolocation, from signal design to the question about whether echolocation is used during foraging. It remains to be seen if bird echolocation is as sophisticated as that of tongue-clicking rousette bats. Bird echolocation performance appears to be superior to that of blind humans using signals of notable similarity. However, no apparent specializations have been found so far in the birds' auditory system (from middle ear to higher processing centers). The advent of light-weight recording equipment and custom software for examining signals and reconstructing flight paths now provides the potential to study the echolocation behavior of birds in more detail and resolve such issues.

## Introduction

In 1794, Lazzaro Spallanzani reported that blinded bats oriented in complete darkness, and, except for the fluttering of their wings, did so silently. Almost 20 years later, Alexander von Humboldt entered a cave in Venezuela and heard resident Oilbirds (*Steatornis caripensis*, von Humboldt, 1817) clicking noisily as they flew around in the cave that served as the birds' day roost. Had the two men corresponded, the behavior of von Humboldt's Oilbirds might have provided Spallanzani with the clue required to solve his famous bat puzzle, and brought ahead the study of animal sonar (echolocation) by about 135 years. We now know that Spallanzani's “silent” bats and von Humboldt's clicking birds use the same sensory mechanism, negotiating their surroundings via echo-feedback from self-emitted sounds. One key difference being that most echolocating bats operate using ultrasonic frequencies above the human hearing range (>20 kHz) and undetectable by eighteenth and nineteenth century technology. Since Griffin's discovery of biosonar using ultrasonic sound above the range of human hearing [reviewed in Griffin (1958)], it has become evident that toothed whales also use echolocation to negotiate their underwater habitat and detect and track their prey (Kellogg and Kohler, 1952; Norris et al., 1961).

Animal sonar is not, however, synonymous with ultrasound. Echolocation signals of several bat and odontocete species include frequencies well below the 20 kHz limit of human hearing (Leonard and Fenton, 1984; Rydell and Arlettaz, 1994; Møhl et al., 2003). Echolocation based in part or entirely on audible signals has also been demonstrated in three species of Old World fruit bats (*Rousettus aegyptiacus, R. leschenaulti, and R. amplexicaudatus)* within the otherwise non-echolocating family Pteropodidae (Möhres and Kulzer, 1956; Novick, 1958). Certain tenrecs (Tenrecidae) from Madagascar (Gould, 1965), several species of shrew (Soricidae) (Gould et al., 1964; Buchler, 1976; Tomasi, 1979; Forsman and Malmquist, 1988; Siemers et al., 2009) and some blind people (Supa et al., 1944; Griffin, 1958; Thaler et al., 2011) also echolocate with signals of frequencies below 20 kHz.

**Figure 1:**
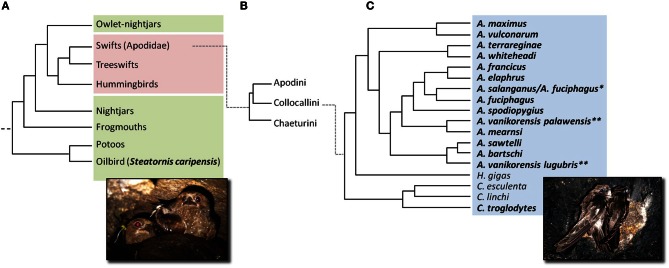
**Composite phylogeny based on three separate studies showing relationships between (A) Apodiformes (hummingbirds and swifts—purple) and Caprimulgiformes (nightjars and allies—green) (Hackett et al., 2008), (B) swifts (Apodidae) (Päckert et al., 2012), and (C) swiftlets (Collocallini, blue) (Thomassen et al., 2005).** Swiftlets are monophyletic and comprise three genera: *Aerodramus* spp., *Hydrochous gigas*, and *Collocalia* spp. (Thomassen et al., 2005). Twenty-six swiftlet species are currently recognized (Chantler et al., 1999; Thomassen, 2005). Nine species (*A. brevirostris*, *A. hirundinaceus*, *A. infuscatus*, *A. inquietus*, *A. leucophaeus*, *A. nuditarsus*, *A. orientalis, A. papuensis*, and *A. unicolor*) were not included in the shown phylogeny and the placement of A. fuciphagus^*^ and A. vanikorensis^**^ was ambiguous. Echolocating species appear in bold. Echolocation has been confirmed for 16 swiftlet species; *H. gigas*, *C. esculenta* and *C. linchi* do not echolocate. Echolocation abilities of remaining species are uncertain. Photographs by Signe Brinkløv: **(A)** Oilbirds (*Steatornis caripensis*) photographed on nest at Dunstan's Cave, Asa Wright Nature Centre, Trinidad (2012), **(C)** Indian Swiftlets (*Aerodramus unicolor*) photographed on nest in a railway tunnel near Pattipola, Sri Lanka (2012).

The only non-mammalian echolocators discovered to date are two groups of birds (Figure 1), the Oilbird (Steatornithidae, Caprimulgiformes) and several species of swiftlets (Apodiformes, Apodidae, Collocalliini, *Aerodramus* spp. and *Collocalia troglodytes*). Given the benefits of biosonar under conditions of poor visibility, seals and owls had been proposed as possible echolocators (e.g., Poulter, 1963; Renoulf and Davies, 1982) but neither echolocate (Crafford and Ferguson, 1999; Schusterman et al., 2000). Why echolocation has evolved in some disparately related groups, but not in others, remains a tantalizing question, suggesting that ecological factors play a greater role in its evolution than physiological constraints and opportunities.

Echolocation research over the last 25 years has focused on the biosonar systems of bats and odontocetes. The few published studies of bird echolocation provide important neuroethological insight and background (Griffin and Suthers, 1970; Fenton, 1975; Konishi and Knudsen, 1979; Griffin and Thompson, 1982; Thompson and Suthers, 1983; Coles et al., 1987; Thomassen et al., 2004; Thomassen and Povel, 2006) but also emphasize that there are many unresolved questions. We suggest that bird echolocation, while almost certainly not as specialized as that of bats and whales, holds the untapped potential for basic research on echolocation using sounds audible to humans, as well as for practical applications such as acoustic monitoring for conservation and management of these often vulnerable birds. Light-weight, state-of-the art field technology now available for the study of bat sonar should be readily applicable to the study of bird echolocation and should help to overcome the challenge of working in remote settings.

Here we review the sensory ecology of echolocating birds, emphasizing several outstanding questions. We consider the design of the birds' echolocation signals, their hearing, and their foraging and roosting behavior. We also speculate about the function and evolution of echolocation in birds and compare it to its use in bats and toothed whales. We further consider why most groups of echolocators, including the birds, use click-type signals rather than the frequency-modulated, often multi-harmonic, signals used by today's laryngeal echolocating bats.

## Ecology of echolocating birds

### Oilbird ecology

Oilbirds (Figure 1) roost in natural caves, primarily in tropical forest across NW South America and Trinidad from sea level to 3400 m (Thomas, 1999). Most other caprimulgids (e.g., night hawks and nightjars) are predominantly insectivorous, crepuscular foragers relying on vision to detect and track prey. Oilbirds are nocturnal fruit-eaters, preferentially eating fruits of palms (Palmaceae), laurels (Lauraceae), and incense (Burseraceae). They swallow the fruits whole (up to 6 × 3 cm), digest the pericarp, and regurgitate the seeds (Snow, 1961, 1962; Bosque et al., 1995). A recent GPS-tracking study from Caripe in Venezuela reported that the birds often spend the day outside their roosting cave, sitting quietly in trees (Holland et al., 2009). Detailed accounts of Oilbird ecology are found in Snow (1961, 1962) and Roca (1994).

Briefly, Oilbirds are large (ca. 400 g, body length 45 cm beak-tip of tail, wing span up to 1 m) and capable of slow, maneuverable flight, with estimated flight speeds of 0.5–7 m/s, and of hovering in narrow spaces (Snow, 1961). Like other caprimulgids, Oilbirds have large eyes relative to their head size (Figure 1) but smaller than those of owls (Warrant, 2008). Oilbirds and owls have similar, low F-numbers (ratio of focal length to pupil diameter) indicating good visual sensitivity (Warrant, 2008). Remarkably, Oilbirds possess a banked retina with rod receptors arranged in a 3-layered structure, conferring a much higher rod to cone ratio than in owls (Warrant, 2008) with higher rod density (~1,000,000 mm^−2^) than any other vertebrate (Martin et al., 2004). This may confer Oilbirds greater visual sensitivity in low-light conditions than owls. Whether this highly sensitive vision trades off spatial resolution remains to be determined (Warrant, 2008). Oilbirds appear to depend primarily on vision whenever possible as evidenced by observations that the incidence of sonar click emissions declines on brightly moonlit nights or in the presence of artificial light sources (Griffin, 1953; Konishi and Knudsen, 1979; Signe Brinkløv and John M. Ratcliffe, pers. obs.). Tapeta lucida occur in the eyes of some caprimulgids (Nicol and Arnott, 1974) but apparently not in Oilbirds (Martin et al., 2004). Oilbirds have large, heavily innervated olfactory organs, suggesting that sense of smell plays an important role in foraging. The birds' own musty odor may play a role in individual recognition (Snow, 1961). Like other caprimulgids, Oilbirds have long rictal bristles around the beak, which may have a close-range tactile function (Snow, 1961).

### Swiftlet ecology

Swiftlets are monophyletic (Thomassen et al., 2003, 2005; Price et al., 2004; Hackett et al., 2008) comprising approximately 26 species (Apodiformes, Apodidae). Swiftlets are found across the Indo-Pacific region, from the Seychelles and Mascarenes in the Indian Ocean to Tahiti, Mo'orea and the Marquesas in the South Pacific (Chantler et al., 1999; Thomassen, 2005). Numerous subspecies have been identified but swiftlet phylogenetic relationships are not fully resolved (Thomassen et al., 2005). This reflects a lack of distinguishing morphological and nest characteristics as well as incomplete phylogenetic sampling (Chantler et al., 1999). An attempt to use echolocation as a discriminative character to split swiftlets into echolocating (*Aerodramus*) and non-echolocating (*Collocalia* and *Hydrochous*) genera (Brooke, 1970, 1972; Medway and Pye, 1977) was refuted because Pygmy Swiftlets (*C. troglodytes*) also echolocate (Price et al., 2004). Only further research will determine whether or not the *Aerodramus* and *Collocalia* genera are justified and will be maintained (Thomassen et al., 2005).

Swiftlets are much smaller (~10 g) than Oilbirds and all species have long, narrow wings (Chantler et al., 1999), characteristic of the typical fast flight of other apodids (Lack, 1956; Videler et al., 2004). Swiftlets are mainly diurnal foragers and hunt small insects on the wing (Chantler et al., 1999; Fullard et al., 2010). At night they typically roost in nests located on the walls of natural caves or mines and tunnels, but intriguingly, there are some published observations of nocturnal activity, including feeding, by some swiftlet species outside their cave roosts (Fullard et al., 1993; Chantler et al., 1999; Price et al., 2005). Swiftlet nests are constructed and glued in place with the birds' own saliva and nests of several species are collected for “birds' nest soup,” a billion dollar industry fueled by human demand (Chantler et al., 1999).

Similar to the situation for bats within the *Rousettus* genus (Giannini and Simmons, 2003), not all swiftlets echolocate. Echolocation has been confirmed in some species, dismissed in others, and for some species we simply do not know. While *Hydrochous gigas*, *Collocalia esculenta*, and *C. linchi* (Figure 1) do not echolocate (Cranbrook and Medway, 1965; Medway and Wells, 1969; Fenton, 1975), at least 16 other swiftlet species do (*C. troglodytes*, *Aerodramus elaphrus*, *A. francicus*, *A. salanganus*, *A. bartschi* (Price et al., 2004); *A. vanikorensis*, (Griffin and Suthers, 1970); *A. brevirostris*, *A. fuciphagus*, *A. maximus*, *A. vulcanorum*, *A. terrareginae* (Thomassen et al., 2004); *A. sawtelli* (Fullard et al., 1993); *A. spodiopygius* (Griffin and Thompson, 1982); *A. papuensis* (Price et al., 2005); *A. hirundinaceus*, *A. unicolor* (Chantler et al., 1999; Signe Brinkløv, pers. obs.). Echolocation abilities of additional species (*A. nuditarsus*, *A. inquietus*, *A. leucophaeus*, *A. whiteheadi*, *A. pelewensis*, *A. orientalis*, *A. mearnsi*, and *A. infuscatus*) are assumed, but remain unconfirmed (Chantler et al., 1999). Swiftlets have relatively large eyes for their body size and they appear to use vision even in low-light conditions (Thomassen, 2005). We were unable to find quantitative data on the visual acuity of swiftlets.

## Biosonar sound production physiology in echolocating birds

Birds produce their echolocation signals in the syrinx, the vocal organ specific to birds and found near to where the trachea forks into the lungs. The production mechanism for echolocation signals has been studied in one species of swiftlet with a tracheo-bronchial syrinx (Suthers and Hector, 1982; Thomassen, 2005), and in the Oilbird, which has a bronchial and bilaterally asymmetric syrinx (Griffin, 1944; Suthers and Hector, 1985). No direct observations have been made of the syringes of either Oilbirds or swiftlets, and the following description may need revision in light of more recent work on bird vocal production physiology (Goller and Larsen, 1997; Elemans et al., 2004; Thomassen, 2005).

With these caveats in mind, phonation (clicks and other acoustic signals) in both groups is driven by subsyringeal pressure, initiated during expiration, and controlled by two antagonistic muscle pairs. Contraction of an extrinsic muscle pair (*mm. sternotrachealis*) folds the external tympaniform membranes into the syrinx (or the two half-syringes in Oilbirds) lumen toward the internal tympaniform membranes. The membranes are then set into vibration by the expiratory airflow. In Oilbirds, clicks are actively terminated by contraction of the single pair of intrinsic syringeal muscles (*mm. broncholateralis*). In contrast, the social vocalizations of Oilbirds are terminated passively by relaxation of the sternotrachealis muscles (Suthers and Hector, 1985). Swiftlets lack intrinsic syringeal muscles and terminate their clicks by contraction of extrinsic tracheolateralis muscles (Suthers and Hector, 1982; Thomassen, 2005). Most species of echolocating swiftlet produce single clicks as well as double clicks (two single clicks in quick succession, as described below). The pause between two clicks within a click-pair may be caused by a brief blocking of airflow through the syrinx as the external and internal tympaniform membranes touch. Single clicks appear to arise when the membranes are pulled together before the expiratory airflow generates enough pressure to initiate vibration of the membranes (Suthers and Hector, 1982). Both sides of the swiftlet syrinx appear able to contribute to each member of a click-pair; that is, birds can still emit double clicks even if one side of the syrinx is plugged (Suthers and Hector, 1982).

## Biosonar signal design in echolocating birds

**Figure 2:**
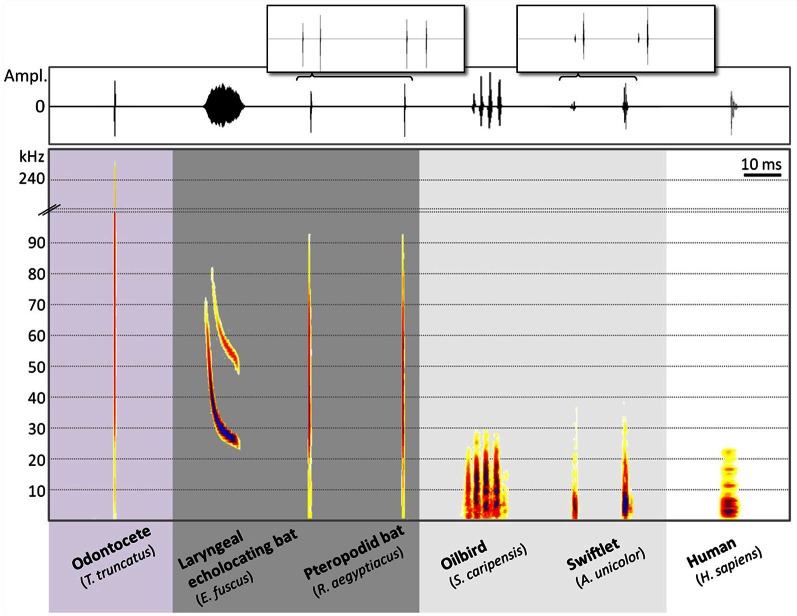
**Composite waveform (top) and spectrogram (bottom) of echolocation signals from 6 vertebrate species: common bottlenose dolphin (*Tursiops truncatus*), sample rate (*f*s) = 500 kHz; laryngeal echolocating bat (*Eptesicus fuscus*), *f*s = 250 kHz; tongue-clicking pteropodid bat (*Rousettus aegyptiacus*), *f*s = 250 kHz; Oilbird (*Steatornis caripensis*), *f*s = 75 kHz; swiftlet (*Aerodramus unicolor*), *f*s = 250 kHz and echolocating blind human subject (*Homo sapiens*), *f*s = 48 kHz.** Top inserts both have total time scales of 300 ms and illustrate the double clicks often emitted by echolocating *Rousettus* spp. and most echolocating swiftlet species. Bat and bird recordings made by Signe Brinkløv, dolphin recording courtesy of Magnus Wahlberg, human recording courtesy of Cynthia Moss. Spectrograms were created in BatSound v. 4 using an FFT size of 256, except for those from *R. aegyptiacus* and *S. caripensis*, for which an FFT size of 128 was used. All spectrograms were made using 98% overlap. Colors indicates relative amplitude going from low (light color) to high (darker color). Note the interrupted frequency scale between 100 and 230 kHz. Waveform amplitudes have all been normalized to the same level.

Echolocation behavior involves the same operating principles across animal groups, namely extracting information about the immediate surroundings from returning echoes of one's own signals. However, vocal physiology, mechanisms of sound production, and signal design differ notably among echolocators. The term click is loosely used to describe acoustic signals that are short and do not exhibit any structured changes in frequency over time. Birds, odontocetes, shrews, tenrecs, and echolocating rousette bats use click-type biosonar signals. Contrarily, laryngeal echolocating bats produce acoustic signals characterized by structured changes in frequency over time, such as downward sweeps (Figure 2). In our discussion of bird echolocation signals, we will follow Pye's definition of clicks as “broadband impulse sounds with no clearly defined coherent ‘carrier’ frequency, no evidence of frequency modulation and an amplitude pattern that is rapid and transient” (Pye, 1980). We will use “click” to define the basic signal unit of bird echolocation and “click burst” to describe two or more clicks produced in rapid succession.

### Echolocation signal design in oilbirds

The first description of Oilbird sonar emissions was based on field recordings of naturally behaving birds flying within a cave (90 m from entrance) at Caripe, Venezuela (Griffin, 1953). Signals from sequences where only one bird was detected on the microphone were described as stereotyped and readily audible to humans at a distance up to 180 m from the bird. Each click consisted of only a few sound waves, and thus was of very brief duration (ca. 1 ms), with most energy between 6 and 10 kHz (Table 1). Notably, clicks were not emitted at a regular rate, but in bursts of 2–6+ clicks, with nearly constant within-burst click intervals of 2.6 ms and little within-burst variation (Griffin, 1953).

**Table 1:** **Summary of Oilbird (*Steathornis caripensis*) echolocation click parameters described in previous literature**.

**References**	**Click parameters**		**Recording site**	**Recording condition**	**System frequency response**
	**Duration (ms)**	**Frequency (kHz)**			
Griffin, 1953	1	6–10	Field Venezuela	Inside cave	Within ±6 dB 50–15,000 Hz
Konishi and Knudsen, 1979	>20	1.5–2.5	Aviary Trinidad	Birds hovering	Flat 50–20,000 Hz
Suthers and Hector, 1985	40–50	No data	Laboratory Trinidad	Handheld birds, blindfolded	Flat 100–40,000 Hz

*Despite similar frequency responses across recording systems, data for click duration and frequencies with most energy are noticeably different. Griffin (1953) reported that a 2 kHz high pass filter was used in the analysis of some recordings but that such a filter was implemented only after verification that the unfiltered recordings had no appreciable energy components below 2 kHz*.

**Figure 3:**
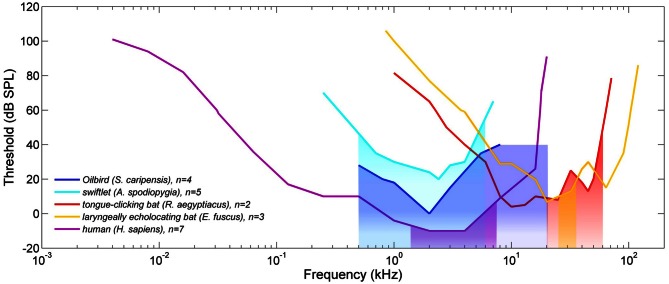
**Comparative audiograms for 5 vertebrates, all of which are capable of some form of echolocation.** Audiograms shown are visually estimated averages derived from previous experiments with Oilbirds (*Steatornis caripensis*) (Konishi and Knudsen, 1979), one swiftlet species (*Aerodramus spodiopygia*) (Coles et al., 1987), one tongue-clicking pteropodid bat species (*Rousettus aegyptiacus*) (Koay et al., 1998), one laryngeal echolocating bat species (*Eptesicus fuscus*) (Koay et al., 1997) and humans (*Homo sapiens*) (Jackson et al., 1999). Audiograms of *R. aegyptiacus*, *E. fuscus* and *H. sapiens* were obtained from behavioral experiments, whereas thresholds from *S. caripensis* and *A. spodiopygia* were based on neurophysiological data from anaesthized birds. Note that relative threshold differences should not be directly compared due to differences in experimental conditions, e.g., different ambient noise levels. Colored blocks correspond to the frequency range where echolocation signals of each group have most energy (measured as −15 dB bandwidth—frequency range 15 dB down from either side of the spectrum peak—of a single click per species), for example the red block is the −15 dB bandwidth of a *R. aegyptiacus* click. The recording used for bandwidth measurements of human echolocation clicks was provided by C. Moss and the −15 dB bandwidth of *A. spodiopygia* was estimated from Figure 3B in Coles et al. (1987). Remaining bandwidths were measured from recordings made by Signe Brinkløv.

Konishi and Knudsen (1979) reported that Oilbird signal energy was unevenly distributed from 1 to 15 kHz, with most energy from 1.5 to 2.5 kHz, coincident with the birds' most sensitive area of hearing (Konishi and Knudsen, 1979). The auditory threshold curve, derived from cochlear evoked potentials, showed maximum sensitivity at 2 kHz, with a roughly 20 dB decline per octave for higher frequencies, indicating that Oilbirds should be deaf, or at least largely insensitive, to sounds above 6 kHz (Figure 3). Konishi and Knudsen (1979) included obstacle avoidance experiments revealing that Oilbirds successfully detect and avoid disks of ≥20 cm diameter but may have failed to detect disks ≤10 cm diameter. However, discs with diameters ≤20 cm were presented in an array where individual disks were spaced at 5 times the chosen disc diameter. This means that trials with discs ≤10 cm likely affected the ability of the Oilbirds to negotiate a course through such an array, as the inter-disc spaces (≤50 cm) were only half of the birds' wingspan. As in bats and whales, an increase in signal repetition rate was noted prior to avoidance manoeuvres (Konishi and Knudsen, 1979).

Suthers and Hector (1983) reported that Oilbirds acoustically detected obstacles as small as 3.2 cm in diameter using signals with most energy at 0.5–3.0 kHz. They suggested that the birds used either continuous pulsatile signals (durations of 40–80 ms) or, occasionally, much shorter pulses emitted at repetition rates ranging from only a few every second to 12 s^−1^ (Suthers and Hector, 1985). From handheld birds, Suthers and Hector (1983) estimated signal intensity as ~100 dB SPL rms at 20 cm to 1 m distance. There are no published quantitative estimates of signal intensity in free-flying Oilbirds and we do not know if the birds can adjust the intensity of their signals, as do laryngeal echolocating bats and toothed whales.

### Echolocation signal design in swiftlets

Echolocation has been confirmed in 16 species of swiftlets (Chantler et al., 1999) and existing descriptions of swiftlet echolocation signals are more congruent, even across recording conditions, than those for Oilbirds (Table 2). Swiftlet clicks are composed of frequencies completely within the human auditory range, with most energy between 1 and 10 kHz.

**Table 2:** **Summary of swiftlet echolocation click parameters as described in the literature**.

**References**	**Species**	**Click parameters**						**Recordingsite**	**Recording condition**	**System frequency response**
		**Click duration (ms)**		**Intra-pair interval (ms)**	**Inter-pair interval (ms)**	**Click frequency (kHz)**				
		**1st**	**2nd**			**1st**	**2nd**			
Griffin and Suthers, 1970	*A. vanikorensis*	Few	4–8	Several	48–358	5–8	5–8	Dark chamber	Flying birds	within ±3 dB 700–12,000 Hz
								Papua		
								New Guinea		
Fenton, 1975	*A. hirundinacea*	−	4–8	−	48–250	4–8	4–8	Cave	Free-flying birds	Within +2/−6 dB 70–13,000 Hz
								Papua		
								New Guinea		
Suthers and Hector, 1982	*A. spodiopygia*	3	>3	18–25	−	2–8	2–8	Dark chamber	Hovering birds	Flat 100–37,000 Hz or 100–75,000 Hz
								Australia		
Coles et al., 1987	*A. spodiopygia*	3–8	4–6	18	> 40	3–8	4–6	Dark chamber	Tethered bird attempting flight	Not described
								Australia		
Fullard et al., 1993	*A. sawtelli*	1–3	−	−	107–186	6–8		Cave Atiu	Free-flying birds	within ±2 dB 500–15,000 Hz
Thomassen and Povel, 2006	*A. brevirostris*	2	3	12	−	3–5	3–6	Various localities and contexts		−
	*A. elaphrus*	2	3	12		2–5	2–6		
	*A. fuciphagus*	3	3	12		3–6	3–7		
	*A. maximus*	6	7	11		3–6	2–7		
	*A. salanganus*	4	5	14		3–6	3–7		
	*A. terrareginae*	2	3	18		3–6	2–7		
	*A. vanikorensis*	2	3	17		2–4	2–5		
	*A. vulcanorum*	2	4	14		2–7	1–10		

*All click parameters are rounded to nearest whole number. (−) indicates that data was not described in the literature source or, in the case of A. sawtelli, that no values exist because this species only produces single clicks*.

With notable exceptions, most swiftlet species emit both single and double clicks (Thomassen et al., 2004). Double clicks, or click-pairs, are emitted more frequently than single clicks (up to 75% of the time) and so close together that they, as the click-bursts of Oilbirds, sound like a single sound to human ears (Griffin and Suthers, 1970). Each click within a pair lasts 1–8 ms, with the second often of higher amplitude (Griffin and Suthers, 1970; Suthers and Hector, 1982; Coles et al., 1987). Clicks in a pair are separated by 11–25 ms (Table 2).

Swiftlet clicks have been described as highly stereotyped, varying little in design regardless of situation (Thomassen and Povel, 2006). However, swiftlets increase click repetition rate when facing complex challenges, such as approaching obstacles (Griffin and Suthers, 1970; Coles et al., 1987) or their nests (Signe Brinkløv, pers. obs. of *A. unicolor* in railway tunnels). Fullard et al. (1993) found that birds emitted higher repetition rates when entering caves than when exiting caves or flying from closed to more open space. Meanwhile, no context-dependent changes were found in signal frequency (Fullard et al., 1993), as compared to the adaptive, context-dependent changes in signal frequency found in many laryngeal echolocating bats.

## Current knowledge of the echolocation abilities of birds

### Echolocation and hearing abilities of oilbirds

Oilbirds have only a single middle ear bone in each ear (as opposed to the three found in mammals), a simple cochlea (Martin, 1990), and thus, like other birds, are expected to be insensitive to frequencies above 10 kHz (Dooling et al., 2000). As noted above, Oilbirds emit conspicuous echolocation signals at frequencies well within the human hearing range and little to no energy above 20 kHz. However, it remains unclear whether most frequency content falls below 5 kHz (Konishi and Knudsen, 1979), or above 5 kHz as described in the earlier field study (Griffin, 1953). Konishi and Knudsen (1979) argued that main frequency content at 6–10 kHz, as reported by Griffin (1953), would result in a mismatch between emitter and receiver. However, Konishi and Knudsen (1979) displayed data points on Oilbird auditory sensitivity up to but not beyond 8 kHz. None of the studies described above seem limited by the frequency range of the recording systems used (Table 1) and so the upper limit of sound frequencies tested by Konishi and Knudsen (1979) was apparently based on the reasonable assumption that Oilbirds do not hear frequencies above 8 kHz. Konishi and Knudsen (1979) also suggest that Oilbirds exhibit little or no directional hearing at frequencies up to 4 kHz and beyond, as predicted by the size of the birds' heads and lack of any external ear structures. While Griffin's (1953) work was done in the field, Konishi and Knudsen's (1979) descriptions are from captive animals. If Oilbirds can change the frequency content of their clicks by shifting signal energy to higher frequencies in the presence of loud ambient low frequency noise, this might occur more often in the field than in captivity.

Existing descriptions of echolocation signal parameters from Oilbirds also reveal discrepancies concerning signal duration (Table 1) and raise questions about how clicks in general are defined by bioacousticians. Griffin (1953) described Oilbird biosonar signals as having a minimum duration of 1 ms, thus referring to a click as the smallest subunit within a burst of sonar emissions. Konishi and Knudsen (1979) used “click' to describe each >20 ms burst of pulses, reasoning based on their recordings that each burst comprises a complex waveform with pulsatile elements rather than a series of discrete pulses. They noted increases in repetition rate between rather than within burst units as birds approached a variety of obstacles. They also argued that because each burst, rather than each burst subunit (i.e., click), is registered as a single, coherent unit by the human ear, by extension they would be registered as a single sound at the bird's more simple ear. Suthers and Hector (1985; their Figure 5) also referred to each click as a burst of several amplitude peaks rather than the subunits within each burst. The number of subunits within a burst varies (Griffin, 1953; Signe Brinkløv, pers. obs.), but whether this variation is of any functional significance to the birds is unknown. The well rounded, if conflicting, data set on Oilbird echolocation makes this species especially attractive for future integrative lab and field-based studies.

### Echolocation and hearing abilities of swiftlets

Swiftlet clicks appear to have most energy over a 1–10 kHz frequency range. Based on rule of thumb calculations, the birds should only detect objects ≥34 mm diameter, but can apparently detect objects as small as 6.3 mm diameter (metal rods) at levels above chance (Griffin and Suthers, 1970; Griffin and Thompson, 1982). Corroborating this, Smyth and Roberts (1983) reported a detection threshold of 10–20 mm, while Fenton (1975) found that *A. hirundinacea* detected vertical rods down to 10 mm diameter and potentially even smaller. These data suggest that swiftlets receive useful echo information via the higher frequency portions of their clicks, even though these components contain less energy. However, for this to be plausible the birds must hear, at least to some extent, higher frequencies. This is not supported by data from single neuron recordings from the midbrain auditory nucleus of *Collocalia spodiopygia*, which indicate best frequency thresholds from 0.8 to 4.7 kHz (Coles et al., 1987).

Whatever the ultimate size limit of object detection by swiftlet biosonar, observations of increased click repetition rates from birds approaching their nests in the wild (Fullard et al., 1993; Signe Brinkløv, pers. obs.) suggest that swiftlets use echolocation to locate their nests. And, because swiftlet nests are 50–100 mm in diameter (Coles et al., 1987; Chantler et al., 1999), even a conservative detection size threshold would indicate that the nest itself should be readily detectable by swiftlet echolocation.

### Single and double swiftlet biosonar clicks: a West-East transition?

*A. sawtelli*, endemic to Atiu, one of the Cook Islands, only emits single clicks, giving rise to the hypothesis of an evolutionary West-East transition from double clicks to the obligate emission of single echolocation clicks (Fullard et al., 1993, 2010). However, Thomassen et al. (2004) reported that several relatively western species of swiftlets can also emit single clicks. Conversely, *A. vanikorensis* in the more centrally located Phillipines and New Guinea appears to emit only double clicks (Thomassen et al., 2004).

Whether single and double clicks serve specific, even separate functions that are correlated to certain behaviors is also unknown, as is whether swiftlets can actively control which type is emitted. Interestingly, although assumed to echolocate, we are unaware of scientific accounts of echolocation in the Polynesian Swiftlet, *A. leucophaeus*, at the far eastern geographic distribution of swiftlets. *A. leucophaeus* is missing from recent attempts to resolve the controversial swiftlet phylogeny but ostensibly includes three subspecies found on Tahiti, Mo'orea, and the Marquesas in French Polynesia (Chantler et al., 1999). More knowledge about the genetic relationship between *A. leucophaeus* and the geographically close single click emitter *A. sawtelli*, along with information about the nature of *A. leucophaeus* echolocation clicks, could help elucidate why some swiftlets only emit single clicks and possibly the underlying functional reasons for the use of single and double clicks.

Egyptian rousettes (*R. aegyptiacus*, Pteropodidae) use double clicks to point their sound beam to the right and left of a target to trade localization over detection (Yovel et al., 2010). Rousette bats echolocate using tongue clicks and this means of echolocating contrasts with the situation in laryngeal echolocating bats, which direct their sonar beam with high precision directly at the target (Jakobsen and Surlykke, 2010). It would be interesting to see whether the double clicks of swiftlets function like those of *Rousettus*.

## Echolocation for orientation, echolocation for food detection?

Echolocating birds use clicks dominated by low frequencies (Konishi and Knudsen, 1979; Coles et al., 1987), limiting their ability to detect small targets. A target reflects echoes only if its cross section is at least roughly one-third as large as the wavelengths impinging on it (Pye, 1980; Jakobsen et al., 2013). Therefore, bird echolocation clicks are not suited for detection of smaller objects such as insect prey <2–3 cm in diameter. Although echolocating birds appear to lack the highly specialized and flexible echolocation abilities of laryngeal echolocating bats and toothed whales they are clearly adept at maneuvering and locating their nests within the dark interior of their cave roosts.

Several anecdotal observations suggest that Oilbirds occasionally echolocate outside caves and around fruiting palm trees (Konishi and Knudsen, 1979; Suthers and Hector, 1985). Snow (1961) reported that he never heard clicks from Oilbirds feeding at night. Staff at the Asa Wright Nature Center in Trinidad provided us with contradictory reports indicating that Oilbirds do click while flying around fruiting palms (Signe Brinkløv, pers. comm.). As Oilbirds eat fruit that is considerably larger than the insect prey of swiftlets (Snow, 1961; Bosque et al., 1995) and often visit trees with a conspicuous shape (e.g., palms), the use of echolocation to find food remains an enticing possibility.

One of us (M. Brock Fenton) has spent considerable time listening for echolocation clicks from swiftlets on Papua New Guinea (*A. hirundinacea*) and in Australia (*A. spodiopygia*) and never heard clicks from night-flying birds except as they returned to their roosts. Notably, however, Atiu Swiftlets (*A. sawtelli*) and Papuan Swiftlets (*A. papuensis*) click not only in their caves but also outside at night, apparently while hunting insect prey in low light (Fullard et al., 1993; Chantler et al., 1999; Price et al., 2005). In swiftlets, echolocation may thus be more advanced in some species than others, but this is highly speculative. If so, the relationship between two click/one click flexibility and the use of echolocation outside the cave would be one area to explore. Oilbirds and swiftlets both orient visually when ambient light conditions are sufficient, as indicated by the absence of echolocation sounds altogether under such conditions and suggested by their oversize eyes relative to other birds. However, the absence of data on light levels taken concurrently with acoustic recordings make it unclear under exactly what conditions the birds should be expected to rely on echolocation over vision.

## Echolocation in a social context

Inside their roosts, echolocating Oilbirds and swiftlets must deal with a host of reverberations from cave surfaces as well as a cacophony of clicks from conspecifics. Besides orientation, bird echolocation signals may serve a role in communication. Laryngeal echolocating bats react to the feeding buzzes emitted by con- and hetero-specifics moments before contact with an airborne insect (Gillam et al., 2007; Übernickel et al., 2013), and change their echolocation behavior when flying in groups as opposed to alone (Obrist, 1995; Ratcliffe et al., 2004; Brinkløv et al., 2009).

In addition to echolocation clicks, Oilbirds and swiftlets produce a range of more tonal signals (Suthers and Hector, 1985; Thomassen and Povel, 2006). For example, Oilbird social squawks resemble a prolonged click burst, including up to 20+ subunits, and are often emitted as several birds fly together (Suthers and Hector, 1985). Such signals likely serve a communicative function to birds flying in close proximity (e.g., as agonistic “honks” to prevent collision, Signe Brinkløv, pers. obs.), analogous to social functions suggested for bat buzzes (i.e., call rates >100calls/s) emitted outside the context of prey-capture (Bayefsky-Anand et al., 2008). Moreover, both Oilbirds and swiftlets appear to forage socially, as indicated by observations of birds arriving at feeding locations and returning to caves in groups of 2 or more individuals (Snow, 1961; Signe Brinkløv, pers. obs.). Swiftlets should be able to maintain visual contact during their daytime foraging bouts, but for nocturnal Oilbirds, biosonar signals may facilitate social cohesion in flight.

There is enough inter-specific variation in swiftlet biosonar clicks to render them species-specific, primarily based on inter-specific variation of maximum click frequency (Thomassen and Povel, 2006). It is plausible then that swiftlet echolocation clicks could be used in conspecific recognition, potentially of relevance where several species have overlapping geographical distributions and may either share or compete for access to caves. However, the social signals of swiftlets are also species-specific (Thomassen and Povel, 2006) and may serve equally well or better for this and other purposes. On a similar note, the morphological asymmetry of the Oilbird syrinx may allow for individual recognition during vocal communication. Individual differences in vocal tract asymmetry have been suggested as a means for Oilbirds to distinguish echoes originating from their own echolocation signals from those clicks and echoes originating from their roostmates (Suthers and Hector, 1988).

## Why click?

Many species of non-echolocating swiftlets and swifts (Apodidae) are acoustically conspicuous to human observers. Two examples are the “screaming” parties of Common Swifts on the wing (*Apus apu*s; Lack, 1956) and the conspicuous flight chirps of Chimney Swifts (*Chaetura pelagica;* Bouchard, 2005). Indeed, the syringes of most non-Oscine birds (e.g., Oilbirds and swiftlets) are well-suited to producing a wide range of acoustic signals (Suthers and Hector, 1985). Why then, do Oilbirds and swiftlets use clicks for echolocation? As Buchler and Mitz (1980) noted, there is no obvious reason why two signals with the same power spectra, one a click, the other a frequency-modulated signal, should differ in their basic utility in echolocation. If anything, single-sweep, frequency-modulated signals may be advantageous, allowing the echolocator to produce a longer signal, with more overall energy, in which a particular frequency is essentially time-stamped (Simmons and Stein, 1980).

We propose that echolocating birds use click-type signals for echolocation because they are short in duration, permitting detection of objects even at very short distances (i.e., with no overlap between signal and echo). At the same time click-type signals do not require the laryngeal specializations observed in bats necessary to produce a sufficiently short frequency-modulated signal. In the non-echolocating Chimney Swifts, none of the frequency-modulated and/or harmonic signals reported by Bouchard (2005) would be short enough to serve as an effective echolocation signal in a cave roost. Additionally or alternatively, clicks may be more effective biosonar signals for detection of objects at greater distances because they may be (i) less energetically expensive to produce using the syrinx and (ii) louder than other signal designs using the same energy input. We note that despite several attempts to uncover any morphological and neurological specializations, none have yet been found in the syringeal morphology, hearing abilities, middle ear morphology or higher processing centers (auditory nuclei) of Oilbirds or echolocating swiftlets that set them apart from non-echolocating birds (Konishi and Knudsen, 1979; Thomassen, 2005; Iwaniuk et al., 2006).

## Evolution of bird echolocation

A recent phylogenomic study of the birds embeds swiftlets within what appears to be the paraphyletic Caprimulgiformes, the avian order that includes Oilbirds (Hackett et al., 2008). Nevertheless, the most parsimonious evolutionary scenario consists of three independent originations of syringeal echolocation in birds, once in the precursor to Oilbirds and twice within the swiftlets (Figure 1). Both groups use echolocation to gain access to roosting sites and nests in caves and deep gorges, where they may be protected from some predators. This common ecological variable may have provided evolutionary impetus for the multiple appearances of echolocation within the clade. An analogous connection between cave-dwelling and use of echolocation seems to be present in rousette bats (Giannini and Simmons, 2003). One avenue of future research would be investigations of the species-specific relationships between the visual systems, presence or absence of echolocation, and preferred light-level of the cave roost within an evolutionary context using the comparative method. Information about the ontogeny of echolocation is at present also completely unknown.

Echolocation almost certainly originated independently in Apodiformes and Caprimulgiformes and likely evolved independently within two distinct lineages of swiftlets (Price et al., 2005; Thomassen et al., 2005). The inaccessibility of many species of swiftlets and resulting lack of genetic and acoustic data means that the evolutionary pathways of swiftlet echolocation remain to be unravelled. Increased molecular sampling and systematic documentation of swiftlet echolocation abilities will be necessary to further resolve their phylogenetic history. Such research would help to clarify species limits, answer questions about the evolution of obligate single click emitting species and address the predominance of those species that produce both double and single biosonar clicks.

Most echolocating bats forage only at night (Neuweiler, 1984), spending the day resting in their roosts. Echolocating swiftlets, like the vast majority of birds, are diurnal foragers (Chantler et al., 1999). Thus, despite their use of cave roosts and similarities in feeding ecology (i.e., the capture of flying insects on the wing) (Fenton, 1975), swiftlets and similar-sized insect-eating bats are not likely to compete with one another directly, due to temporal separation of foraging activities. Similarly, there is no evidence that either echolocating bats or swiftlets feed on one another. Oilbirds and rousette bats exploit a similar niche, albeit on different continents. Interestingly, both Oilbirds and rousette bats are nocturnal frugivores, and both use click-type echolocation and dark roosts during the day (Griffin et al., 1958; Snow, 1961). In the New-World tropics, where Oilbirds and a number of smaller frugivorous New World leaf-nosed bats (Phyllostomidae) overlap both spatially and temporally when foraging, there appears to be very little overlap in fruit preference between these groups. Oilbirds consume large fruits, often with large seeds that are later regurgitated (Snow, 1962), while phyllostomid bats are much smaller and feed preferentially on fruits with small seeds that are chewed or expelled while eating (Wendeln et al., 2000; Mello et al., 2011).

## Future research steps

Further studies of the echolocation systems of birds will be valuable additions to the ever-expanding and progressive field of bat and toothed whale echolocation research. State-of-the-art lightweight field equipment (e.g., multi-microphone arrays) and custom-designed computational software should provide better quality recordings of biosonar signals from Oilbirds and swiftlets. Experiments could be designed to compare signals of birds flying in different contexts, for example, field versus captivity, open space versus cave interior and multiple versus single birds, to help resolve current uncertainties about signal design. Further, such recordings should help identify *who* says *what*, *when*, and *where* even in complex situations where several birds are flying together and provide useful clues about echolocation in a social context.

The highly specialized echolocation systems of toothed whales and laryngeal echolocating bats have provided and continue to provide fascinating insights into the mammalian auditory system and active sensory processes in animals across taxa. By comparison, echolocation in birds has received almost no attention. This is perhaps because we have implicitly regarded bird biosonar as unsophisticated and, thus, less interesting. Perhaps, less cynically, it is simply because bats are found everywhere, save past the tree-line and on a few isolated Oceanic islands, while echolocating birds are far less wide-spread and in general more difficult to gain access to than are bats.

Deployment of portable tags with hydrophones and accelerometers has contributed greatly to the understanding of toothed whale acoustic behavior in deep waters where the animals roam beyond visual inspection (Madsen et al., 2005; Johnson et al., 2007; Jensen et al., 2011). Corresponding on-board archival microphone tags would be ideal to assess the level of any active and adaptive control over sonar signal characteristics in birds, clarify the potential role of bird echolocation in the context of in-flight social interactions and allow us to determine if Oilbirds echolocate while foraging. In-flight GPS recorders have already been used to track movements of Oilbirds in the field (Holland et al., 2009) and their large size makes Oilbirds ideal subjects for the first acoustic tagging study of echolocating birds. Further, direct endoscopic visualization of syringeal mechanisms is now possible (Goller and Larsen, 1997), as are *in vitro* neuromuscular preparations to study the biomechanic mechanisms involved in avian and mammalian sound production (Elemans et al., 2004, 2011). Such techniques could be put to use in better understanding biosonar click production in Oilbirds and swiftlets.

The tongue-clicking pteropodid bat *R. aegyptiacus* uses echolocation to detect and discriminate objects better than previously suspected (Yovel et al., 2011). Echolocation in birds may be similarly underappreciated. Moreover, a deeper understanding of echolocation in birds, rousette bats, and shrews and tenrecs would have its own rewards. Echolocation by blind people is now more common and better understood, and comparisons to non-human echolocators using similar click-type signals may help us learn more about and improve human biosonar. In a broader sense, understanding animal biosonar across taxa will undoubtedly reveal similarities and differences across different groups of animals that have independently evolved biosonar systems with respect to all aspects of their biology, from ecology and evolution, to the neurophysiology and biomechanics of sound production and echo processing.

### Conflict of interest statement

The authors declare that the research was conducted in the absence of any commercial or financial relationships that could be construed as a potential conflict of interest.

## References

[B1] Bayefsky-Anand S. Skowronski M. D. Fenton M. B. Korine C. Holderied M. W. (2008). Variations in the echolocation calls of the European free-tailed bat. J. Zool. 275, 115–123 10.1111/j.1469-7998.2008.00418.x

[B2] Bosque C. Ramirez R. Rodriguez D. (1995). The diet of the Oilbird in Venezuela. Ornitologia Neotropical 6, 67–80

[B3] Bouchard J. (2005). The Role of Acoustic Signals in Flying Chimney Swifts, Chaetura Pelagica. M.Sc. thesis, Department of Biology, University of Western Ontario, London, ON.

[B4] Brinkløv S. Kalko E. K. V. Surlykke A. (2009). Intense echolocation calls from two ‘whispering’ bats, *Artibeus jamaicensis* and *Macrophyllum macrophyllum* (Phyllostomidae). J. Exp. Biol. 212, 11–20 10.1242/jeb.023226 19088206

[B5] Brooke R. K. (1970). Taxonomic and evolutionary notes on the subfamilies, tribes, genera and subgenera of the swifts (Aves: Apodidae). Durb. Mus. Nov. 9, 13–24

[B6] Brooke R. K. (1972). Generic limits in old world Apodidae and Hirundinidae. Bull. Br. Ornithol. Club 92, 53–57

[B7] Buchler E. R. (1976). The use of echolocation by the wandering shrew (*Sorex vagrans*). Anim. Behav. 24, 858–873 10.1016/S0003-3472(76)80016-4

[B8] Buchler E. R. Mitz A. R. (1980). Similarities in design features of orientation sounds used by simpler, nonaquatic echolocators, in Animal Sonar Systems, eds Busnel R.-G. Fish J. F. (New York, NY: Plenum Press), 871–874

[B9] Chantler P. Wells D. R. Schuchmann K. L. (1999). Family Apodidae (swifts), in Handbook of the Birds of the World, Vol. 5 *Barn Owls to* Hummingbirds, eds del Hoyo J. Elliott A. Sargatal J. (Barcelona: Lynx), 388–457

[B10] Coles R. B. Konishi M. Pettigrew J. D. (1987). Hearing and echolocation in the Australian Grey Swiftlet, *Collocalia spodiopygia*. J. Exp. Biol. 129, 365–371

[B11] Crafford D. Ferguson J. W. H. (1999). Why do Grass Owls (*Tyto capensis*) produce clicking calls? J. Raptor Res. 33, 134–142

[B12] Cranbrook E. O. Medway L. (1965). Lack of ultrasonic frequencies in the calls of swiftlets (*Collocalia* spp.). Ibis 107, 258 10.1111/j.1474-919X.1965.tb07305.x

[B13] Dooling R. J. Lohr B. Dent M. L. (2000). Hearing in birds and reptiles, in Comparative Hearing: Birds and Reptiles, eds Dooling R. J. Popper A. N. Fay R. R. (New York, NY: Springer), 308–359

[B14] Elemans C. P. H. Spierts I. L. Y. Müller U. K. van Leeuwen J. L. Goller F. (2004). Superfast muscles control dove's trill. Nature 431, 146 10.1038/431146a 15356620

[B15] Elemans C. P. H. Mead A. F. Jakobson L. Ratcliffe J. M. (2011). Superfast muscles set maximum call rate in echolocating bats. Science 333, 1885–1888 10.1126/science.1207309 21960635

[B16] Fenton M. B. (1975). Acuity of echolocation in *Collocalia hirundinacea* (Aves: Apodidae), with comments on the distributions of echolocating swiftlets and molossid bats. Biotropica 7, 1–7

[B17] Forsman K. A. Malmquist M. G. (1988). Evidence for echolocation in the common shrew, *Sorex araneus*. J. Zool. Soc. Lond. 216, 655–662 10.1111/j.1469-7998.1988.tb02463.x

[B18] Fullard J. H. Barclay R. M. R. Thomas D. W. (1993). Echolocation in free-flying Atiu Swiftlets (*Aerodramus sawtelli*). Biotropica 25, 334–339

[B19] Fullard J. H. Barclay R. M. R. Thomas D. W. (2010). Observations on the behavioural ecology of the Atiu Swiftlet *Aerodramus sawtelli*. Bird Cons. Int. 20, 385–391

[B20] Giannini N. P. Simmons N. B. (2003). A phylogeny of megachiropteran bats (Mammalia: Chiroptera: Pteropodidae) based on direct optimization analysis of one nuclear and four mitochondrial genes. Cladistics 19, 496–511 10.1016/j.cladistics.2003.09.002 34905855

[B21] Gillam E. H. Ulanovsky N. McCracken G. F. (2007). Rapid jamming avoidance in biosonar. Proc. R. Soc. B. 274, 651–660 10.1098/rspb.2006.0047 17254989 PMC2197216

[B22] Goller F. Larsen O. N. L. (1997). A new mechanism of sound generation in songbirds. Proc. Natl. Acad. Sci. U.S.A. 94, 14787–14791 9405691 10.1073/pnas.94.26.14787 PMC25115

[B23] Gould E. (1965). Evidence for echolocation in the Tenrecidae of Madagascar. Proc. Am. Phil. Soc. 109, 352–360

[B24] Gould E. Negus N. C. Novick A. (1964). Evidence for echolocation in shrews. J. Exp. Zool. 156, 19–37 14189919 10.1002/jez.1401560103

[B25] Griffin D. R. (1944). Echolocation by blind men, bats and radar. Science 29, 589–590 10.1126/science.100.2609.589 17776129

[B26] Griffin D. R. (1953). Acoustic orientation in the Oilbird, *Steatornis*. Proc. Natl. Acad. Sci. U.S.A. 39, 884–893 16589349 10.1073/pnas.39.8.884 PMC1063873

[B27] Griffin D. R. (1958). Listening in the Dark. New Haven, CT: Yale University Press

[B28] Griffin D. R. Novick A. Kornfield M. (1958). The sensitivity of echolocation in the fruit bat, *Rousettus*. Biol. Bull. 115, 107–113

[B29] Griffin D. R. Suthers R. A. (1970). Sensitivity of echolocation in cave swiftlets. Biol. Bull. 139, 495–501 5494235 10.2307/1540368

[B30] Griffin D. R. Thompson T. (1982). Echolocation by cave swiftlets. Behav. Ecol. Sociobiol. 10, 119–123 10.1007/BF00300171

[B31] Hackett S. J. Kimball R. T. Reddy S. Bowie R. C. K. Braun E. L. Braun M. J. (2008). A phylogenomic study of birds reveals their evolutionary history. Science 320, 1763–1768 10.1126/science.1157704 18583609

[B32] Holland R. A. Wikelski M. Kümmeth F. Bosque C. (2009). The secret life of Oilbirds: new insights into the movement ecology of a unique avian frugivore. PLoS ONE 4:e8264 10.1371/journal.pone.0008264 20016844 PMC2788423

[B33] Iwaniuk A. N. Clayton D. H. Wylie D. R. W. (2006). Echolocation, vocal learning, auditory localization and the relative size of the avian auditory midbrain nucleus (MLd). Behav. Brain Res. 167, 305–317 10.1016/j.bbr.2005.09.015 16256215

[B34] Jackson L. L. Heffner R. S. Heffner H. E. (1999). Free-field audiogram of the Japanese macaque (*Macaca fuscata*). J. Acoust. Soc. Am. 106, 3017–3023 10573911 10.1121/1.428121

[B35] Jakobsen L. Surlykke A. (2010). Vespertilionid bats control the width of their biosonar sound beam dynamically during prey pursuit. Proc. Natl. Acad. Sci. U.S.A. 107, 13930–13935 10.1073/pnas.1006630107 20643943 PMC2922241

[B36] Jakobsen L. Ratcliffe J. M. Surlykke A. (2013). Convergent acoustic field of view in echolocating bats. Nature 493, 93–96 10.1038/nature11664 23172147

[B37] Jensen F. H. Marrero Perez J. Johnson M. P. Aguilar Soto N. Madsen P. T. (2011). Calling under pressure: short-finned pilot whales make social calls during deep foraging dives. Proc. R. Soc. B. 278, 3017–3025 10.1098/rspb.2010.2604 21345867 PMC3158928

[B38] Johnson M. Hickmott L. S. Aguilar Soto N. Madsen P. T. (2007). Echolocation behaviour adapted to prey in foraging Blainville's beaked whale (*Mesoplodon densirostris*). Proc. R. Soc. B. 275, 133–139 10.1098/rspb.2007.1190 17986434 PMC2596185

[B39] Kellogg W. N. Kohler R. (1952). Reactions of the porpoise to ultrasonic frequencies. Science 116, 250–252 10.1126/science.116.3010.250 17742549

[B40] Koay G. Heffner H. E. Heffner R. S. (1997). Audiogram of the big brown bat (*Eptesicus fuscus*). Hear. Res. 105, 202–210 10.1016/S0378-5955(96)00208-0 9083817 10.1016/s0378-5955(96)00208-0

[B41] Koay G. Heffner R. S. Heffner H. E. (1998). Hearing in a megachiropteran fruit bat (*Rousettus aegyptiacus*). J. Comp. Psychol. 112, 371–382 10.1037/0735-7036.112.4.371 9861710

[B42] Konishi M. Knudsen E. I. (1979). The Oilbird: hearing and echolocation. Science 204, 425–427 10.1126/science.441731 441731

[B43] Lack D. (1956). Swifts in a Tower. London: Methuen

[B44] Leonard M. L. Fenton M. B. (1984). Echolocation calls of *Euderma maculatum* (Vespertilionidae): use in orientation and communication. J. Mammal. 65, 122–126

[B45] Madsen P. T. Johnson M. Aguilar de Soto N. Zimmer W. M. X. Tyack P. (2005). Biosonar performance of foraging beaked whales (*Mesoplodon densirostris*). J. Exp. Biol. 208, 181–194 10.1242/jeb.01327 15634839

[B46] Martin G. R. (1990). Birds by Night. London: T. and A. D. Poyser

[B47] Martin G. R. Rojas L. M. Ramírez Y. McNeil R. (2004). The eyes of Oilbirds (*Steatornis caripensis*): pushing a the limits of sensitivity. Naturwissenschaften 91, 26–29 10.1007/s00114-003-0495-3 14740100

[B48] Medway L. Pye J. D. (1977). Echolocation and the systematics of swiftlets, in Evolutionary Ecology, eds Stonehouse B. Perrins C. (Baltimore, MD: University Park Press), 225–238

[B49] Medway L. Wells D. R. (1969). Dark orientation by the Giant Swiftlet *Collocalia gigas*. Ibis 111, 609–611 10.1111/j.1474-919X.1969.tb02570.x

[B50] Mello M. A. R. Marquitti F. M. D. Guimaraes P. R. Jr. Kalko E. K. V. Jordano P. de Aguiar M. A. M. (2011). The missing part of seed dispersal networks: structure and robustness of bat-fruit interactions. PLoS ONE 6:e17395 10.1371/journal.pone.0017395 21386981 PMC3046224

[B51] Møhl B. Wahlberg M. Madsen P. T. Heerfordt A. Lund A. (2003). The monopulsed nature of sperm whale clicks. J. Acoust. Soc. Am. 114, 1143–1154 12942991 10.1121/1.1586258

[B52] Möhres F. P. Kulzer E. (1956). Über die Orientierung der Flughund (Chiroptera-Pteropodidae). Z. Vergl. Physiol. 38, 1–29

[B53] Neuweiler G. (1984). Foraging, echolocation and audition in bats. Naturwissenschaften 71, 446–455

[B54] Nicol J. A. C. Arnott H. J. (1974). Tapeta lucida in the eyes of goatsuckers (Caprimulgidae). Proc. R. Soc. B. 187, 349–352 4154455 10.1098/rspb.1974.0079

[B55] Norris K. S. Prescott J. H. Asa-Dorian P. V. Perkins P. (1961). An experimental demonstration of echo-location behavior in the porpoise, *Tursiops truncatus* (Montagu). Biol. Bull. 120, 163–176

[B56] Novick A. (1958). Orientation in Paleotropical bats. II. Megachiroptera. J. Exp. Zool. 137, 443–462 10.1002/jez.1401370305 13587875

[B57] Obrist M. K. (1995). Flexible bat echolocation: the influence of individual, habitat and conspecifics on sonar signal design. Behav. Ecol. Sociobiol. 36, 207–219 10.1007/BF00177798

[B58] Päckert M. Martens J. Wink M. Feigl A. Tietze D. T. (2012). Molecular phylogeny of Old World swifts (Aves: Apodiformes, Apodidae, *Apus* and *Tachymarptis*) based on mitochondrial and nuclear markers. Mol. Phylogen. Evol. 63, 606–616 10.1016/j.ympev.2012.02.002 22361213

[B59] Poulter T. C. (1963). Sonar signals of the sea lion. Science 139, 753–755 10.1126/science.139.3556.753 17829121

[B60] Price J. J. Johnson K. P. Clayton D. H. (2004). The evolution of echolocation in swiftlets. J. Avian Biol. 35, 135–143 10.1111/j.0908-8857.2004.03182.x

[B61] Price J. J. Johnson K. P. Bush S. E. Clayton D. H. (2005). Phylogenetic relationships of the Papuan Swiftlet *Aerodramus papuensis* and implications for the evolution of avian echolocation. Ibis 147, 790–796 10.1111/j.1474-919X.2005.00467.x

[B62] Pye J. D. (1980). Echolocation signals and echoes in air, in Animal Sonar Systems, eds Busnel R.-G. Fish J. F. (New York, NY: Plenum Press), 309–353

[B63] Ratcliffe J. M. ter Hofstede H. M. Avila-Flores R. Fenton M. B. McCracken G. F. Biscardi S. (2004). Conspecifics influence call design in the Brazilian free-tailed bat, *Tadarida brasiliensis*. Can. J. Zool. 82, 966–971 10.1139/z04-074

[B64] Renoulf D. Davies M. B. (1982). Evidence that seals may use echolocation. Nature 300, 635–637 10.1038/300635a0 7144913

[B65] Roca R. L. (1994). Oilbirds of Venezuela: Ecology and Conservation. Cambridge, MA: Publications of the Nuttall Ornithological Club

[B66] Rydell J. Arlettaz R. (1994). Low-frequency echolocation enables the bat *Tadarida teniotis* to feed on tympanate insects. Proc. R. Soc. Lond. B 257, 175–178 10.1098/rspb.1994.0112 7972162

[B67] Schusterman R. J. Kastak D. Levenson D. H. Reichmuth C. J. Southall B. L. (2000). Why pinnipeds don't echolocate. J. Acoust. Soc. Am. 107, 2256 10.1121/1.428506 10790051

[B68] Siemers B. M. Schauermann G. Turni H. von Merten S. (2009). Why do shrews twitter? Communication or simple echo-based orientation. Biol. Lett. 5, 593–596 10.1098/rsbl.2009.0378 19535367 PMC2781971

[B69] Simmons J. A. Stein R. A. (1980). Acoustic imaging in bat sonar: echolocation signals and the evolution of echolcoation. J. Comp. Physiol. A. 135, 61–84

[B70] Smyth D. M. Roberts J. R. (1983). The sensitivity of echolocation by the Grey Swiftlet *Aerodramus spodiopygius*. Ibis 125, 339–345 10.1111/j.1474-919X.1983.tb03119.x

[B71] Snow D. W. (1961). The natural history of the oilbird, *Steatornis caripensis*, in Trinidad, W. I. Part 1. General behavior and breeding habits. Zoologica 46, 27–48

[B72] Snow D. W. (1962). The natural history of the oilbird, *Steatornis caripensis*, in Trinidad, W. I. Part 2. Population, breeding ecology and food. Zoologica 47, 199–221

[B73] Supa M. Cotzin M. Dallenbach K. M. (1944). “Facial Vision”: the perception of obstacles by the blind. Am. J. Psychol. 57, 133–183

[B74] Suthers R. A. Hector D. H. (1982). Mechanism for the production of echolocating clicks by the Grey Swiftlet, *Collocalia spodiopygia*. J. Comp. Physiol. 148, 457–470 10.1007/BF00619784

[B75] Suthers R. A. Hector D. H. (1983). Mechanisms of sonar pulse production by Oilbirds. J. Acoust. Soc. Am. 74(Suppl. 1), 31 10.1121/1.2020912

[B76] Suthers R. A. Hector D. H. (1985). The physiology of vocalization by the echolocating Oilbird, *Steatornis caripensis*. J. Comp. Physiol. A 156, 243–266 10.1007/BF00610867

[B77] Suthers R. A. Hector D. H. (1988). Individual variation in vocal tract resonance may assist oilbirds in recognizing echoes of their own sonar clicks, in Animal Sonar: Processes and Performance, eds Nachtigall P. E. Moore P. W. B. (New York, NY: Plenum Press), 87–91

[B78] Thaler L. Arnott S. R. Goodale M. A. (2011). Neural correlates of natural human echolocation in early and late blind echolocation experts. PLoS ONE 6:e20162 10.1371/journal.pone.0020162 21633496 PMC3102086

[B79] Thomas B. T. (1999). Family Steatonithidae (Oilbird), in Handbook of the Birds of the World. Vol. 5 Barn Owls to hummingbirds, eds del Hoyo J. Elliott A. Sargatal J. (Barcelona: Lynx), 244–251

[B80] Thomassen H. A. (2005). Swift as Sound – Design and Evolution of the Echolocation System in Swiftlets (Apodidae: Collocaliini). Ph.D. thesis, Leiden University.

[B81] Thomassen H. A. Povel G. D. E. (2006). Comparative and phylogenetic analysis of the echo clicks and social vocalisations of swifts and swiftlets (Aves: Apodidae). Biol. J. Linn. Soc. 88, 631–643 10.1111/j.1095-8312.2006.00648.x

[B82] Thomassen H. A. Djasim U. M. Povel G. D. E. (2004). Echo click design in swiftlets: single as well as double clicks. Ibis 146, 173–174 10.1111/j.1474-919X.2004.00237.x

[B83] Thomassen H. A. den Tex R.-J. de Bakker M. A. G. Povel G. D. E. (2005). Phylogenetic relationships of some swifts and swiftlets; a multi locus approach. Mol. Phylogenet. Evol. 37, 264–277 10.1016/j.ympev.2005.05.010 16006151

[B84] Thomassen H. A. Wiersema A. T. de Bakker M. A. G. de Knijff P. Hetebrij E. Povel G. D. E. (2003). A new phylogeny of swiftlets (Aves: Apodidae) based on cytochrome b DNA. Mol. Phylogenet. Evol. 29, 86–93 10.1016/S1055-7903(03)00066-6 12967609

[B85] Thompson D. B. Suthers R. A. (1983). Acuity of echolocation on the Oilbird, *Steatornis caripensis*. J. Acoust. Soc. Am. 74(Suppl. 1), 31 10.1121/1.2020913

[B86] Tomasi T. E. (1979). Echolocation by the short-tailed shrew *Blarina brevicauda*. J. Mammal. 60, 751–759 10.2307/1380190 750639

[B87] Übernickel K. Tschapka M. Kalko E. K. V. (2013). Selective eavesdropping behaviour in three neotropical bat species. Ethology 119, 66–76 10.1111/eth.12038

[B88] Videler J. J. Stamhuis E. J. Povel G. D. (2004). Leading-edge vortex lifts swifts. Science 306, 1960–1962 10.1126/science.1104682 15591209

[B88a] von Humboldt A. (1817). Mémoire sur le Guacharo de la caverne de Caripe. Recueil d'Obs. de Zool. E d'Anatomie no. 2.

[B89] Warrant E. (2008). Nocturnal vision, in The Senses: A Comprehensive Reference, Vol. 2, eds Masland R. H. Albright T. (New York, NY: Academic Press). 53–86

[B90] Wendeln M. C. Runkle J. R. Kalko E. K. V. (2000). Nutritional values of 14 fig species and bat feeding preferences in Panama. Biotropica 32, 489–501 10.1111/j.1744-7429.2000.tb00495.x

[B91] Yovel Y. Falk B. Moss C. F. Ulanovsky N. (2010). Optimal localization by pointing off axis. Science 327, 701–704 10.1126/science.1183310 20133574

[B92] Yovel Y. Geva-Sagiv M. Ulanovsky N. (2011). Click-based echolocation in bats: not so primitive after all. J. Comp. Physiol. A. 197, 515–530 10.1007/s00359-011-0639-4 21465138

